# The monocyte-macrophage-mast cell axis in dengue pathogenesis

**DOI:** 10.1186/s12929-018-0482-9

**Published:** 2018-11-08

**Authors:** Shu-Wen Wan, Betty A. Wu-Hsieh, Yee-Shin Lin, Wen-Yu Chen, Yan Huang, Robert Anderson

**Affiliations:** 1 School of Medicine, College of Medicine, I-Shou University, Kaohsiung, Taiwan; 2 Graduate Institute of Immunology, College of Medicine, National Taiwan University, Taipei, Taiwan; 3 Department of Microbiology and Immunology, College of Medicine, National Cheng Kung University, Tainan, Taiwan; 4 Center of Infectious Disease and Signaling Research, National Cheng Kung University, Tainan, Taiwan; 5 Department of Microbiology & Immunology, Dalhousie University, Halifax, NS B3H 4R2 Canada; 6 Canadian Center for Vaccinology, Dalhousie University, Halifax, Canada

**Keywords:** Dengue pathogenesis, Mast cell, Monocyte, Macrophage, Vascular leakage

## Abstract

Dengue virus, the causative agent of dengue disease which may have hemorrhagic complications, poses a global health threat. Among the numerous target cells for dengue virus in humans are monocytes, macrophages and mast cells which are important regulators of vascular integrity and which undergo dramatic cellular responses after infection by dengue virus. The strategic locations of these three cell types, inside blood vessels (monocytes) or outside blood vessels (macrophages and mast cells) allow them to respond to dengue virus infection with the production of both intracellular and secretory factors which affect virus replication, vascular permeability and/or leukocyte extravasation. Moreover, the expression of Fc receptors on the surface of monocytes, macrophages and mast cells makes them important target cells for antibody-enhanced dengue virus infection which is a major risk factor for severe dengue disease, involving hemorrhage. Collectively, these features of monocytes, macrophages and mast cells contribute to both beneficial and harmful responses of importance to understanding and controlling dengue infection and disease.

## Introduction

Dengue is the most common mosquito-transmitted viral infectious disease, and therefore represents a major global health threat, especially in tropical and subtropical areas of the world. Over the past several decades, there has been an increase in dengue cases due to many factors, including increased air travel and climate change. It has been estimated that there are 390 million infections per year, of which 96 million cases show clinical manifestations [1]. Dengue virus (DENV) is a member of the Flavivirus genus of the Flaviviridae family and its genome is a single-stranded, positive-sense RNA, which encodes three structural proteins: envelope (E), premembrane/membrane (prM/M) and capsid (C) proteins, and seven nonstructural (NS) proteins [2]. There are at least four serotypes of DENV, all of which can cause disease. Most dengue patients present with dengue fever (DF) including fever, headache, bone pain and skin rash, but some may progress to life-threatening dengue hemorrhagic fever (DHF) or dengue shock syndrome (DSS) with major features of high levels of proinflammatory cytokines, vascular leakage, thrombocytopenia, hemorrhage, and hypotensive shock [3, 4]. There is still a demand for satisfactory vaccines and antiviral drugs. Although there is a licensed vaccine and several ongoing vaccine candidates in clinical trials, they confer only partial protection against DENV infection by some serotypes and may carry the risk of antibody-dependent enhancement (ADE) [5, 6]. The difficulty of eliciting balanced efficacy of neutralizing antibodies against all four DENV serotypes remains a major concern.

Both innate and adaptive immune responses to DENV play significant roles in protection against DENV, but can also elicit pathological responses which may worsen disease [2, 7]. Understanding the mechanisms that regulate immune-mediated protection versus pathogenesis is critical for the development of safe and effective dengue vaccines and therapeutic agents [4, 7]. DENV can perturb vascular endothelium by multiple mechanisms, including vasoactive factors from intravascular cells such as monocytes and lymphocytes, and from extravascular cells such as mast cells and tissue macrophages. Various factors produced by T cells, monocytes, macrophages, and mast cells have been proposed to increase vascular permeability, including tumor necrosis factor (TNF), IL-1β, IL-6, CXCL8 (IL-8), macrophage migration inhibitory factor (MIF), CCL2 (also known as monocyte chemoattractant protein-1, MCP-1), high mobility group box-1 (HMGB-1) and matrix metalloproteinases [4, 8].

Although DENV infects several cell types, monocytes, macrophages and mast cells are major responders to DENV infection by producing potent immunological mediators, including cytokines, chemokines, lipid-derived mediators and more. By virtue of cell surface expressed Fc receptors they can also function as major amplifiers of DENV infection in the presence of subneutralizing levels of antibody by the mechanism of ADE. In this review, we will focus on cells and cellular networks encompassing monocytes, macrophages, and mast cells that can uniquely amplify DENV infection by ADE and modulate pathogenesis by release of cytokines, chemokines, proteases and other factors.

### The monocyte-macrophage-mast cell axis

Monocytes (and monocyte-derived cells including macrophages) as well as mast cells originate from bone marrow common myeloid progenitors (CMPs) by two distinct pathways. Monocytes differentiate from CMP-derived granulocyte/monocyte progenitors (GMPs) while mast cells develop from CMP- or GMP-derived mast cell progenitors (MCPs) which home to peripheral tissues where they progress to mature mast cells [9]. Mature mast cells, like their precursor MCPs, express the stem cell factor receptor CD117 (c-kit) and the high affinity FcεR but differ from MCPs most notably by their high content of granules [10]. While monocytes remain largely in the circulation, monocyte-derived cells (including macrophages) as well as mast cells are resident in the tissues. All three cell types are potent producers of cytokines, chemokines and other factors some of which affect vascular integrity. Their locations either within or in close proximity to blood vessels as well as their potent innate immune responses to DENV infection allows them to function as an “axis” in regulating the fine balance between virus replication/suppression and disease.

### Monocytes and DENV

Peripheral blood mononuclear cells, particularly monocytes have long been recognized as major targets of DENV infection and amplification [11–14], especially in the presence of low levels of dengue-specific antibody. The dramatic enhancement by dengue antibody of DENV replication in monocytes and certain other cells is known as ADE [15, 16]. DENV comprises four serotypes (designated 1–4) against each of which the respective homotypic antiserum is much more effective than heterotypic antiserum in virus neutralization. ADE is hypothesized to contribute to heightened dengue disease severity and is believed to arise upon sequential infection of an individual with two different DENV serotypes in which antibody produced against the first serotype enhances infection of the second. The formation of virus-antibody complexes in such individuals gives rise to increased virus replication in cells bearing Fc receptors (eg. monocytes and a select number of other cells), triggering amplified virus- and immune-mediated pathogenic effects. In concordance with the ADE hypothesis is a study showing higher numbers of FcγRII bearing DENV-infected monocytes in DHF compared to DF patients [14]. Single nucleotide polymorphisms in the FcγRII gene are also associated with altered susceptibility to severe dengue disease [17].

ADE of DENV infection has been differentiated into intrinsic and extrinsic components, denoting intra- and extracellular events respectively [18, 19]. Extrinsic ADE is believed to result from increased virus binding and internalization via virus-antibody complex ligation of Fc receptors. Intrinsic ADE, which results from signaling through ligated FcRs, is postulated to suppress antiviral responses, selectively enhance cytokine production (particularly IL-10) and enhance virus replication [19]. Additional or alternative mechanisms may also be involved [20].

Infection of monocytes by DENV is dependent on monocyte phenotype, as defined by relative expression levels of certain protein markers which are associated with differentiation or activation status [14, 21, 22]. Human monocytes are in fact a heterogeneous population which can be grouped into at least three subsets [23, 24]. One study found DENV infection predominantly in one subset of monocytes expressing CD14, CD32, CD86 and CD11c [14]. Another study reported upregulation of CD14 and CD16 in DENV-infected blood monocytes which mediate B cell to plasmablast differentiation and production of IgM and IgG [22]. However, another study reported no increase in blood CD14^+^ CD16^+^ monocytes [25]. A correlation between DENV-induced monocyte activation and severity of disease has been reported [14].

DENV infection of monocytes triggers the release of numerous immunological factors, some of which modulate the function of other cells, particularly vascular endothelial cells. Endothelial cells are activated by TNF released by antibody-enhanced DENV-infected monocytes [26]. Circulating TNF levels are altered in severely afflicted dengue patients [27–29] and TNF is a crucial factor in DENV-induced hemorrhage in a mouse model [30]. Moreover, human genetic studies of cytokine gene polymorphisms highlight a strong role for TNF in the severity of dengue disease [17].

In addition to TNF, other monocyte-secreted factors can prime or trigger endothelial cell permeability leading to vascular leakage, a major hallmark of severe dengue disease. Other cells including lymphocytes and endothelial cells themselves can further contribute to vascular leakage via the secretion of similar or other vasoactive factors. Which of these factors predominate in triggering dengue-associated endothelial permeability is widely debated, but likely candidates include vascular endothelial growth factor (VEGF), platelet activating factor (PAF), leukotrienes, matrix metalloproteinase-9 (MMP-9), sphingosine-1-phosphate (S1P), DENV NS1 protein (reviewed in [8]) as well as MIF [31] and glycosaminoglycans such as hyaluronic acid and heparan sulfate [32]. The dominant source(s) of these factors awaits definitive identification, but likely includes monocytes, lymphocytes, endothelial cells and platelets. Platelets, which have been shown to undergo marked changes in protein expression during dengue infection [33], may also enhance cyto/chemokine production by DENV-infected monocytes through a contact-dependent mechanism [34].

A picture of complex cellular interplay during dengue infection is beginning to emerge. For example, dengue-infected monocyte-derived dendritic cells are able to activate natural killer (NK) cells which in turn may suppress DENV infection of monocytes by a mechanism involving interferon (IFN)-γ [35]. DENV replication in monocytes may also be suppressed by NK cell activation through a TRAIL-dependent mechanism augmented by type I IFNs [36]. On the other hand, monocyte-derived dermal macrophages bind and internalize DENV into early phagosomes but do not permit virus replication and may therefore have a role in sequestration and early control of the virus. Such DENV-exposed dermal macrophages apparently do not produce IFN-α [37] and therefore likely restrict virus replication in themselves without conferring antiviral resistance on other cells.

Monocyte-derived cells do not always have a protective role in DENV infection. In the mouse model, monocytes which migrate to the virus inoculation site in the dermis and differentiate into dendritic cells may become fresh targets for virus replication in the skin [38, 39].

It is important to note that DENV-induced cellular (including monocytic) infiltration into the skin is not generalized, but rather is restricted to the site of virus inoculation as indicated by studies on mice [38, 39] as well as cynomolgus macaques [25]. Although limited, studies on humans also indicate a lack of widespread cellular infiltration into skin even in patients showing severe dengue disease, i.e. DSS [25].

Monocytes and monocyte-derived cells therefore play seemingly synergistic as well as opposing roles in dengue disease. In addition to their important beneficial role in virus clearance by activating T cells in the draining lymph node [40], they may also contribute either negatively or positively to virus replication in the skin.

### Role of apoptosis in DENV-monocyte interactions

Apoptosis of peripheral blood mononuclear cells (PBMCs), including lymphocytes and monocytes as well as phagocytic engulfment of apoptotic cells were noted in children with acute DENV infection [41]. Such apoptosis was proposed to represent a modulating mechanism for both virus replication as well as cell-mediated immune responses [41]. Apoptosis has also been observed in DENV-infected monocyte and/or macrophage cultures [42–46]. Multiple mechanisms of apoptosis induction have been proposed including components of both the intrinsic and extrinsic pathways. Caspase-8 activation and concomitant TNF production has been reported in DENV-infected monocyte-like U937 cells [43]. Caspase-1 has been implicated in both IL-1β production and pyroptosis in DENV-infected monocytes [47]. IL-1β is a known activator of endothelial cells and, along with monocyte-produced TNF [26], could contribute to vascular permeability in dengue disease.

### Macrophages and DENV

Jessie et al. [48] identified DENV antigen and RNA in macrophages, multinucleated and reactive lymphoid cells in the spleen, Kupffer cells and sinusoid endothelial cells of liver, and macrophages and endothelial cells in the lungs of human autopsied and biopsied samples. Balsitis et al. [49] identified DENV NS3 protein in phagocytes of spleen and lymph nodes, in alveolar macrophages in the lungs, and in perivascular cells in the brains from dengue autopsy cases. An in vitro study also showed that primary human peripheral monocytes and splenic macrophages are permissive for DENV [50]. The mannose receptor on primary human macrophages that binds to the envelope protein of DENV through its carbohydrate-recognition domain may be responsible for recognition and uptake of the virus [51]. The observation that IL-4 treatment renders human dermal macrophages and dendritic cells isolated from healthy human abdominal skin permissive to DENV infection [52] could be the result of upregulation of the mannose receptor on macrophages by IL-4 [53].

In *Stat1*^*−/−*^ mice infected with DENV, Chen et al. identified CLEC5A as a receptor for DENV [54]. Blocking CLEC5A protected mice from DENV-induced pathology and death [54]. CLEC5A has also been identified as the receptor that mediates DENV-induced IL-1β on GM-CSF-stimulated human monocyte-derived macrophages [55].

In AG129 mice infected subcutaneously with DENV2 (PL046 or mouse-adapted D2S10), viral E and NS1 proteins are detected in F4/80^+^CD11b^+^ macrophages and CD11c^+^ dendritic cells in the spleen and other lymphoid tissues during the early phase of infection [56]. By inoculation of labeled DENV intravenously to AG129 mice, Prestwood et al. [57] found that macrophages, initially in lymphoid tissues, especially in the spleen, are the main virus targets. In the later phase of infection, however, macrophages in non-lymphoid tissues also become targets of DENV replication. In wild-type mice infected by DENV2 through the intradermal route, both macrophages and endothelial cells are targets of the virus [30]. Macrophages are recruited to the vicinity of endothelium during hemorrhage development [58]. Their recruitment and response to the virus has a profound impact on the pathogenesis of hemorrhage [30].

### Cytokine production by macrophages in response to DENV

Human monocyte-derived macrophages infected with DENV in vitro produce TNF, IFN-α, IL-1β, CXCL8 (IL-8), IL-12, CCL3 (MIP-1α) and CCL5 (Regulated on Activation Normal T cell Expressed and Secreted, RANTES) [12]. Autopsy tissues from dengue patients showed elevated levels of IFN-γ and TNF expressing cells in livers, lungs and kidneys [59] and DENV RNA was detected in Kupffer cells producing these two cytokines [59]. The relationship between TNF and hemorrhage is worth noting. An early study in Thai children showed that plasma level of soluble TNF receptor (sTNFR) detected at < 72 h of fever is higher in children who developed DHF than those who had DF and TNF was detectable more often in children with DHF than with DF and children with fever from non-dengue-related illness [60]. TNF, which activates endothelial cells, is also produced by DENV-infected monocytes [26] and mast cells [61]. In a dengue hemorrhagic mouse model, skins obtained from hemorrhagic sites express higher levels of TNF transcripts and protein than that from non-hemorrhagic sites and TNF deficiency impedes DENV-induced hemorrhage development [30]. Immunofluorescence staining of hemorrhage tissues revealed that TNF co-localizes with macrophages and DENV infection of macrophages in vitro also induces TNF production [30]. These data demonstrate that TNF is important in severe dengue in humans as well as hemorrhage development in the mouse.

### Role of apoptosis in DENV-macrophage interactions

Human liver Kupffer cells respond to DENV infection with cytokine production and apoptosis [62]. Although DENV replication is low or absent in cultured Kupffer cells [62], DENV antigen is detectable in Kupffer cells and hepatocytes in human autopsy studies [63]. Phagocytic Kupffer cells may also play a role in clearance of virus-induced apoptotic bodies in infected tissues [64].

Apoptosis is also observed in endothelial cells which are important targets of monocyte/macrophage action. Importantly, TNF and DENV-induced endothelial cell death resulted in alteration of endothelial permeability and pan-caspase treatment reversed its effect [58]. These results demonstrate that infection of endothelial cells by DENV in the presence of TNF changes endothelial permeability through caspase-dependent cell death. In the hemorrhage mouse model, hemorrhage development is accompanied by macrophage recruitment and endothelial cell death [58]. Macrophage production of TNF in the vicinity of endothelium that is infected with DENV may enhance endothelial cell death which contributes to hemorrhage development.

**Fig. 1:**
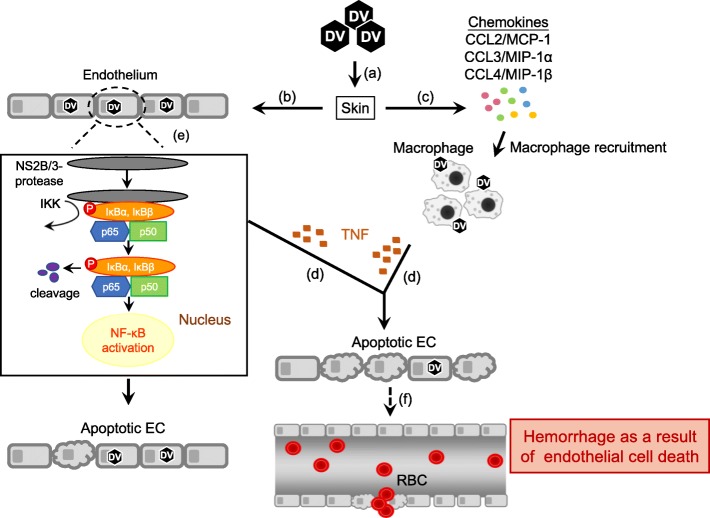
Dengue virus interactions with macrophages and endothelial cells that lead to hemorrhage development. **a** Inoculation by mosquito bite of DENV (DV) into the skin. **b** The virus infects several cell types including endothelial cells (ECs). **c** DENV induces production of chemokines that attract macrophages. **d** DENV stimulates macrophages to produce TNF. **e** DENV NS2B/3 protease interacts with and cleaves cellular IκBα/IκBβ. DENV NS2B/3 protease also activates IKK, which phosphorylates IκBα and IκBβ. IκBα/IκBβ cleavage enables p50 and p65 translocation into the nucleus, thereby activating NF-κB which results in endothelial cell death. **f** The presence of TNF in the microenvironment enhances DENV-primed EC apoptosis. Endothelium damage/increased vascular permeability results in hemorrhage development. Solid arrows represent events that enhance endothelium damage. Dotted arrow indicates an event that is speculated to occur [30, 58, 65]

It is of interest to note that DENV NS2B/3 protease enzymatic activity is critical to DENV-induced endothelial cell death [65]. DENV NS2B/3 protease cleaves host cell IκBα and IκBβ. By inducing IκBα and IκBβ cleavage and IκB kinase activation, enabling p50 and p65 translocation to the nucleus, DENV NS2B/3 protease activates NF-κB which results in endothelial cell death. Injecting DENV NS2B/3 protease packaged in adenovirus-associated virus-9 intradermally to mice induces macrophage infiltration, endothelial cell death and hemorrhage development [65]. Thus, the presence of TNF-producing macrophages near blood vessels contributes to DENV protease-induced endothelial cell death and hemorrhage development. A depiction of the possible events triggered by DENV infection that lead to hemorrhage development is shown in Fig. 1.

### Mast cells and DENV

Mast cells are well known for their involvement in inflammation and allergy but recent studies indicate a broader role in immunological responses [66–69]. The abundance of mast cells at mucosal sites and skin confers on them a sentinel function for the early detection and disposition of invading pathogens. Upon appropriate stimulation, mast cells selectively produce and secrete a variety of mediators including chemokines, cytokines, lipid mediators and granule associated products. Mast cells reside mainly in the tissues and have been shown to associate closely with blood vessels [70] and nerves [71]. Human mast cells can express both FcεRI [72, 73] and some Fcγ receptors including FcγRI [74, 75] and FcγRII [76, 77] and contain FcγRIII mRNA [76].

Mast cells have aroused speculation for many years as to their possible involvement in dengue pathogenesis. Mast cells are located in the skin and mucosa which are the first line of defense against pathogens. In addition to dendritic cells (including Langerhans cells) and macrophages [78], mast cells also encounter DENV early in infection [79]. DHF patients exhibit increased levels of urinary and plasma histamine which is a major granule-associated mediator from mast cells [80, 81]. Levels of mast cell-derived VEGF and proteases are also increased in DSS patients [82]. Furthermore, mast cell-derived chymase also promotes vascular leakage in a DENV-infected mouse model [83]. In vitro studies indicated that antibody-enhanced DENV infection of mast cells selectively induces production of chemokines including CCL3, CCL4 and CCL5 [84, 85], as well as cytokines including IL-6, IL-1β and TNF [61, 86]. TNF produced from antibody-enhanced DENV infection of mast cells as well as of monocytes can trigger endothelial cell activation [26, 61]. These findings suggest that mast cells likely play a role in vascular function as well as leukocyte recruitment during DENV infection.

Most significantly, mast cells are susceptible to antibody-enhanced DENV infection via the mast cell FcγRII [87]. Mast cell responses to antibody-enhanced DENV infection have revealed potent immunoregulatory activities of these cells, including secretion of TNF [61] and the chemokines CCL3, CCL4 and CCL5 [84]. Together with other published reports [67, 68, 88, 89], these studies reinforce the role of mast cells as innate immune effectors in response to a variety of virus infections. Chemokines such as CCL3, CCL4 and CCL5 are important for the trafficking of leukocytes such as monocytes, T cells, and NK cells, all of which are suggested to play important roles in dengue infection. Serum levels of CCL3, CCL4 and CCL5 are altered [90–93] and tissue levels of chemokine-producing cells are elevated [59, 93] in dengue patients. In particular, serum levels of CCL4 are increased in mild dengue and may be of good prognostic value [93].

### Induction of innate immune factors in DENV-infected mast cells

The cellular molecules by which DENV is detected by the innate immune system have been partly characterized. RIG-I or MDA5 have been implicated in the production of CCL5 and CXCL8 by a number of viruses, including DENV as well as viral RNA homologs [85, 94, 95]. Upregulation of RIG-I and MDA5 mRNA has been demonstrated after DENV infection in a rodent mast cell line [79] as well as antibody-enhanced DENV infection of human mast cells [85]. Protein kinase dsRNA dependent (PKR) recognizes dsRNA and can mediate the inhibition of protein translation in response to type I IFNs and DENV dsRNA [96]. Together, all three RNA sensors provide a mechanism by which the innate immune system induces the antiviral response when the host is exposed to DENV.

In addition to the above-noted RNA sensors RIG-I, MDA5 and PKR, mast cells possess a battery of pattern recognition receptors the individual expression of which varies according to the host source and associated tissue or organ [97–100]. Human mast cells express the RNA sensor, Toll-like receptor (TLR)3 [89]. Recognition of viral dsRNA by mast cell TLR3 leads to signaling via TRIF to TBK1/IKKε to activate both IRF-3 and nuclear factor-κB (NF-κB) promoting the production of IFN stimulated genes, cytokines and chemokines. In the case of human mast cell lines HMC-1 and LAD-2 as well as primary peripheral CD34^+^ mast cells, responses to extracellular polyinosinic⋅polycytidylic (polyI:C) were shown to involve upregulation of type I IFNs by RT-PCR [89]. Mast cells activated by polyI:C have also been reported to influence CD8^+^ T cell recruitment [88]. Furthermore, polyI:C-exposed or reovirus-infected mast cells recruit NK cells in a CXCL8-dependent manner [101]. Along with other RNA sensors, TLR3 is also upregulated in antibody-enhanced DENV infection of mast cells [85].

Antibody-enhanced DENV-infected mast cells can produce sufficient amounts of type I IFNs to protect neighboring cells from infection [85]. The upregulation of RNA sensors such as RIG-I and MDA5 appears to be key for the suppression of DENV replication via establishment of the antiviral state [102–104]. The upregulation of PKR in mast cells upon antibody-enhanced DENV infection [85], is also consistent with induction of the antiviral state since protein translation inhibition during DENV infection is dependent on the PKR substrate, eIF2α [96]. The possibility that tissue-resident mast cells can initiate this vital response would therefore allow them to confer type I IFN-mediated protection upon neighboring cells at the tissue site early after virus inoculation.

### Roles of mast cells in DENV clearance and vascular leakage

**Fig. 2:**
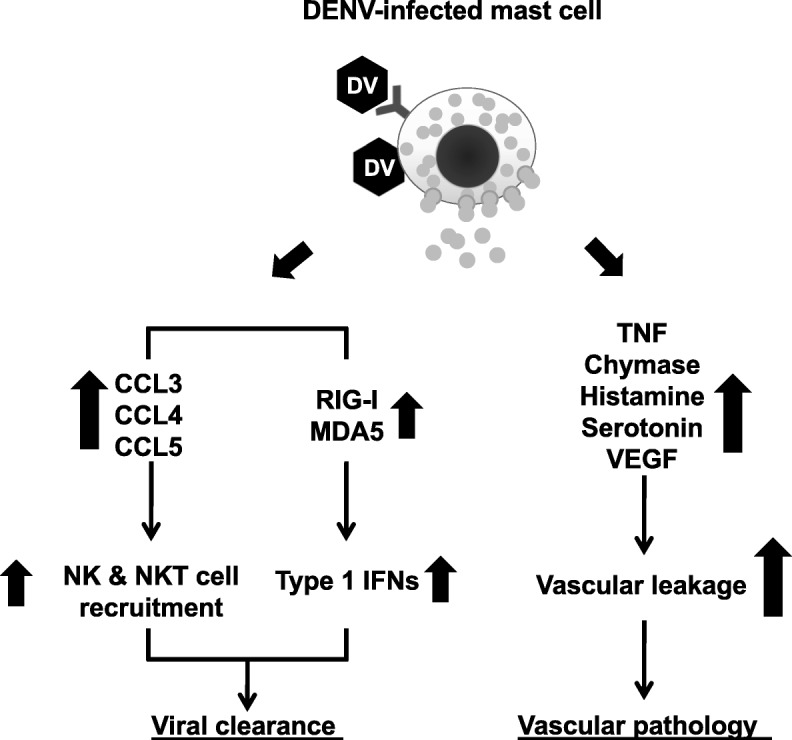
The dual roles of mast cells in dengue infection. Mast cells respond to DENV (DV) infection via RNA sensors (RIG-I and MDA5) which are involved in type I IFN production to inhibit viral replication. DENV-infected mast cells also secrete chemokines including CCL3, CCL4 and CCL5, which recruit NK and NKT cells to help clear the virus. However, if initial control mechanisms fail, the virus may spread to other organs. DENV-infected mast cells in these organs secrete vasoactive products, including TNF, chymase, histamine, and serotonin and VEGF which contribute to vascular permeability

After DENV infection, mast cell-deficient mice showed increased viral burden within draining lymph nodes, compared with wild-type mice. In addition, the recruitment of NK and NKT cells into the DENV-infected skin was dependent on mast cell activation [79]. Such mast cell-dependent immune responses facilitate DENV clearance. Compared to wild-type mice, mast cell-deficient mice showed enhanced DENV infection, CCL2 production and macrophage infiltration at the skin inoculation site, suggesting other mechanisms for the interplay between mast cells and tissue macrophages to modulate DENV replication [105]. Therefore, during the initial stage, mast cells may play crucial roles in immune surveillance for DENV by promoting viral clearance and restricting viral replication (Fig. 2).

**Fig. 3:**
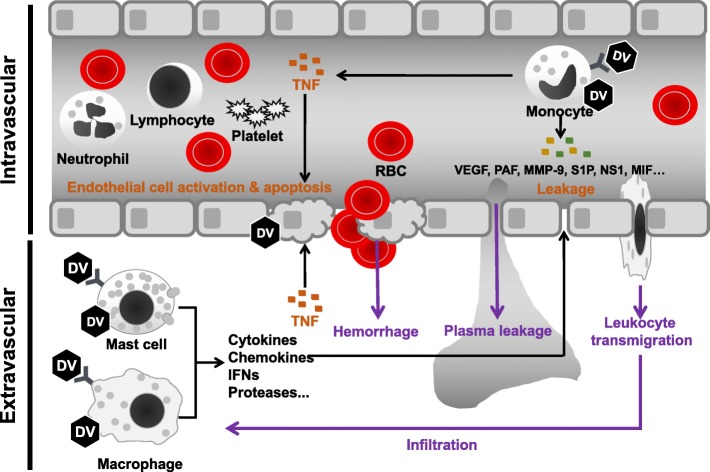
Intra- and extravascular cells in the pathogenesis of dengue. DENV (DV) infection of monocytes triggers the intravascular release of numerous immunological factors to modulate the function of vascular endothelial cells. Besides TNF, other monocyte-secreted factors can prime or trigger endothelial cell permeability leading to vascular leakage and leukocyte transmigration to extravascular tissues. Extravascular mast cells and macrophages are target cells for DENV infection which elicits production of cytokines, chemokines, lipid-derived mediators and proteases which also contribute to endothelial cell permeability. In addition, macrophage production of TNF enhances DENV-infected endothelial cell death which leads to hemorrhage development

Several mast cell-derived mediators, such as tryptase, chymase and VEGF contribute to dengue disease severity [82]. Serum chymase levels could be a predictive biomarker of DHF in pediatric and adult patients [106]. DENV-infected mice show activated degranulated tissue mast cells. as well as elevated systemic levels of various vasoactive products, including chymase, histamine, and serotonin [83]. After DENV infection, mast cell-deficient mice showed significantly reduced vascular permeability compared to mast cell-sufficient controls [83]. Hence, at later stages of systemic infection, mast cells might play other important roles in DENV-induced vascular leakage (Fig. 2). Sub-neutralizing dengue-specific antibodies not only promote DENV infection but also enhance mast cell activation in an FcγR-dependent manner [87, 107]. During secondary DENV infection, antibody-mediated mast cell activation may therefore also contribute to the enhanced vascular pathology in severe dengue (Fig. 3).

The involvement of mast cells in dengue pathogenesis suggests they may be potential therapeutic targets. The mast cell-stabilizing drug, ketotifen, not only improves DENV-induced vasculopathy [83] but also reverses the DENV-induced host response without suppressing memory T cell formation [108]. Furthermore, antibodies against DENV NS1 provide protection in mice against DENV challenge and reduce mast cell degranulation and macrophage infiltration as well as the production of chemokines including CCL2, CCL5, and CXCL10 (IP-10) at local skin DENV infection sites [109].

### Role of apoptosis in DENV-mast cell interactions

Antibody-enhanced DENV-infected mast cell-like KU812 cells show dramatic apoptosis [110]. Interestingly, apoptosis is observed mainly in DENV antigen-negative cells suggesting the involvement of apoptotic mediators produced by DENV-infected cells. Alternatively, apoptosis may be triggered very early in some DENV-infected cells so that cell death occurs prior to appreciable virus replication. Thus, as with monocytes and macrophages, apoptosis of mast cells in DENV infection likely plays a role in regulating mast cell numbers and responses.

## Conclusions

While differing in cellular developmental pathways, monocytes/macrophages and mast cells share intriguing features which come into play in vascular disease triggered by DENV infection. Their potent production of cytokines, chemokines and various vasoactive mediators in response to DENV makes them key orchestrators of some of the pathological vascular changes which occur in severe dengue disease. In particular, their expression of Fc receptors makes them powerful amplifiers of DENV replication as well as of virus-induced innate immune factors some of which act directly on vascular endothelium and others of which regulate the extent of virus replication.

## Abbreviations

CCL: Chemokine (C-C motif) ligand
CMP: Common myeloid progenitor
CXCL: Chemokine (C-X-C motif) ligand
DENV: Dengue virus
DF: Dengue fever
DHF: Dengue hemorrhagic fever (DHF)
dsRNA: double-stranded ribonucleic acid
DSS: Dengue shock syndrome
eIF: eukaryotic initiation factor
GMP: Granulocyte/monocyte progenitor
IFN: Interferon
IL: Interleukin
MIF: Macrophage migration inhibitory factor
MMP: Matrix metalloproteinase
NK: Natural killer
NS1: Non-structural protein 1
PKR: Protein kinase R
TLR: Toll-like receptor
TNF: Tumor necrosis factor
